# Application of latent class analysis in assessing the competency of physicians in China

**DOI:** 10.1186/s12909-017-1039-4

**Published:** 2017-11-13

**Authors:** Zhuang Liu, Yue Zhang, Lei Tian, Baozhi Sun, Qing Chang, Yuhong Zhao

**Affiliations:** 1 School of Public Health, China Medical University, Shenyang, Liaoning China; 2 Research Center for Medical Education, China Medical University, Shenyang, Liaoning China; 3 Department of Clinical Epidemiology, Shengjing Hospital, China Medical University, No.36, Sanhao Street, Heping District, Shenyang, Liaoning Province 110004 People’s Republic of China

**Keywords:** Physicians, Competency, Latent class analysis

## Abstract

**Background:**

The physicians’ competency is an important public health issue around the world. Several international organizations have taken the lead in examining the competencies required to be a physician. The purpose of this study is to identify subgroups of physicians’ competency based upon the importance results of competency evaluation and provide a scientific basis for the qualitative research of the competency of physicians.

**Methods:**

A cross-sectional study was conducted on a large population-based sample in 31 provinces, autonomous regions and municipalities directly under the central government in China. The latent class analysis was performed to identify patterns of physicians’ competency using M-plus software.

**Results:**

In this study, the latent class analysis was adopted to identify the appropriate number of distinct latent classes of physicians’ competency based on eight competency dimensions, and a four-class model best fit the data, which are excellent competency group, lack of professionalism competency group, individual competency driven group, and lack of competency cognitive group. Therefore, 6247 physicians can be divided into four latent classes based on the importance results of competency evaluation, and the number of each class is 5684, 284, 215 and 64, respectively.

**Conclusion:**

These findings suggested that latent class analysis can be used to study the competency of physicians, and four distinct subgroups were identified. Therefore, we can effectively understand the patterns of physicians’ competency, and the health administrative departments could utilize more specific measures according to their different competency subgroups, and providing individualized training schemes in the future training and management of physicians.

**Electronic supplementary material:**

The online version of this article (10.1186/s12909-017-1039-4) contains supplementary material, which is available to authorized users.

## Background

Along with an elevated level of economic development is the improvement of social security systems and advances in medical technology. As well as a diversified public demand for clinical medical services, the demand for clinical medical professionals has also been increasing. Clinical medicine is recognized as the science with dual characteristics of both scientific knowledge systems and practical activities, and the comprehensive ability of physicians is closely associated with the quality of health care. In 2010, the global medical education leaders published a report in the Lancet, which proposed the concept of the third medical education reform: based on the education system and health system, with competency-orientation, to establish occupational ability requirements with specific targets to improve the performance of the whole health system [1].

Around the world, several international organizations have taken the lead in examining the competencies required to be a physician. The United States, Canada and United Kingdom basically completed the competence index system and guidelines for physicians. The USA’s Accreditation Council for Graduate Medical Education (ACGME) has identified 6 domains of clinical competencies for all GME specialties [2, 3]. The Royal College of Physicians and Surgeons of Canada (RCPSC) developed Canadian Medical Education Directives for specialists (CanMEDS) framework, which was a guide to the essential abilities physicians need for optimal patient outcomes [4]. The General Medical Council (GMC) of the United Kingdom has published a version of Good Medical Practice (GMP) for each specialty [5, 6]. In May 2012, Chinese Ministry of Education and Health launched the “excellent physicians training program”, aimed to reform the training mode of clinical medical personnel, and to train high-level, international physicians who would adapt to development and social needs. This is of important strategic significance in deepening the medical system reform and improving the quality of medical professional training [7, 8].

In the research of competency, usually indirect measurement of the latent or implicit competency of the individual can be obtained through the observed and measurable behavior of the samples. Latent class analysis (LCA) is a person-centered statistical method, which assumes that individuals can be grouped into classes with similar patterns of some behaviors according to their response to a set of observed indicators [9, 10]. Therefore, the LCA based on statistical models has important methodological significance for the research of the physicians’ competency [11]. To date, some studies have utilized LCA as a way to describe and identify subgroups of medical students, patients and nurses based on their characteristics and behaviors. For example, the LCA identified three hypothetical classes in the unprofessional behaviors of medical students according to a template of 109 behaviors [12]. Another study suggested four distinct subgroups of complementary medicine users among patients with breast cancer using LCA [13]. Three subgroups were identified for head nurses’ competency based on characteristics of competency self-reported questionnaire [14]. Likewise, researchers have used LCA to explain co-morbidity or to study subtypes based on combinations of emotional and behavioral situations [15–17]. However, these studies did not examine the classes of physicians’ competency. Thus, further research is needed to identify subgroups of physicians based on competency. As a first step in the process of developing competency model, different subtypes of physicians’ competency will have different training and evaluation programs. Therefore, the aim of this study was to explore whether distinct subgroups of physicians’ competency could be defined.

## Methods

### Questionnaires

The questionnaire used was “Chinese physician’s competency questionnaire”, developed by North Medical Education Development Center of China Medical University. The physicians needed to rank the importance of competency items. The questionnaire used a 5-point Likert-scale [18], ranked the items’ importance from 1 (definitely not important), 2 (not important), 3 (neutral), 4 (important) to 5 (definitely important). The questionnaire divided the competency of physicians into eight dimensions, which are: clinical skills and patient care, professionalism, interpersonal communication, master of medical knowledge, teamwork, health promotion and disease prevention, information and management, academic research. There were a total of 103 items, due to the large number of questionnaire items, the current study performed latent class analysis on the average importance scores for each dimension in the questionnaire. The form of the questionnaire is presented in Additional file 1.

### Participants

The approval of the survey was obtained from China Medical University Review Board. Each participant gave verbal and written consent. Participants could retreat from the research at any time. We performed the multi-stage stratified sampling research in 31 cities in all 31 provinces, municipalities and autonomous regions conducted from October 2012 to June 2013. Among the total of questionnaires distributed in the formal survey, 6247 valid questionnaires (89.0%) were collected.

### Data collection

The data was first stratified according to the region and hospital grade, and further stratified according to the title of physicians within the hospital for random sampling. The studied subjects are mostly physicians who have completed 3-years resident standardized training, general rotation or with equivalent qualifications, covering internal medicine, surgery, gynecology and obstetrics, pediatrics and other clinical departments.

Before the investigation, the investigators were uniformly trained with standardized survey language and methods, and the survey questionnaires were numbered. During on-site investigation, the investigators would explain and guide the filling of questionnaires. After the investigation, the questionnaires were reviewed again to sort out incomplete ones. The data input was performed by two trained investigators, with 25% of the questionnaires being randomly selected for double checks to ensure consistency of data entry, and that the data information was true and reliable.

### Data analysis

#### Descriptive analysis and test

The descriptive analysis was used to show the basic characteristics of the physicians in the survey. To compare the effects of different factors on the competency of physicians, the t-test and variance analysis were performed in our research.

#### Basic model of latent class analysis

The LCA was realized by a latent variable analysis model: the latent class model (LCM), which mainly explains and estimates the relationship between observed class variables through latent class variables, and to further maintain the local-independence of the explicit variables. The LCA is a potentially useful characteristics categorization technique for effectively mining class variable data information, which can also make up for the deficiency that category variables can’t be analyzed by other methods, such as factor analysis and structural equation model [19, 20]. It makes up for the deficiency of the structural equation model, which can only deal with the continuous latent variables, and more importantly, the research of the classification latent variables improves the analysis value of the class variables, so that the researchers can more deeply understand the latent impact factors of the class variables through probability [21–23].

The LCA assumes that the relationship between any two observed variables can be explained by the latent variable. Assume that the latent variable X has t latent classes; A, B, C are three explicit variables with a level of I, J, K. The most basic latent class model is:

$$ {\pi}_{ijk}^{ABC}={\sum}_{t=1}^T{\pi}_t^X{\pi}_{it}^{AX}{\pi}_{jt}^{BX}{\pi}_{kt}^{CX} $$

In the equation ${\uppi}_{\mathrm{ijk}}^{\mathrm{ABC}}$ stands for the joint probability of a LCA, ${\uppi}_{\mathrm{t}}^{\mathrm{X}}$ is the probability of observation data belonging to a specific latent variable x, ${\uppi}_{\mathrm{it}}^{\mathrm{AX}}$stands for the conditional probability of a subject belongs to the t-th latent class reacted to the i-th A explicit variable.

#### Model fitting and parameter estimation

The LCA mainly uses the maximum likelihood method to estimate the parameters. The evaluation methods for model fitting include the Pearson test, likelihood ratio chi-square test and information evaluation criteria. AIC (Akaike information criterion) and BIC (Bayesian information criterion) are the most widely used evaluation criteria in LCA selection, both are built based on the likelihood chi-square test and can be used to compare models with a different limitation on parameters, in both of which smaller results mean better fitting. Some researchers have pointed out that the BIC index is more reliable when the sample size is in several thousands. Therefore, the subgroup evaluation in our study is mainly based on the BIC index [24]. In order to more easily explain and understand the LCA, the current study re-categorized the importance evaluation into two relevant competency groups: unimportant and important groups. The unimportant group included the importance evaluation for 1 (unimportance) and 2 (less importance). The important group included the importance evaluation for 3 (general importance), 4 (important) and 5 (very important).

#### Latent classification

After determination of the optimal model, the final step is to assign the observed values into the appropriate latent classes, and to explain the posterior classification properties of the observed value, which is the latent clustering analysis. The LCA was conducted using M-plus V6 [25]. The classification is based on the Bias theory, with the calculation formula as follows:

$$ {\pi}_{tijk}^{XABC}=\frac{\pi_{tijk}^{XABC}}{\sum \limits_{t=1}^T{\pi}_{tijk}^{XABC}} $$

## Results

### Demographic characteristics and comparison of physicians’ competency

The average age of the physicians was 38.98 years (SD = 8.71). The demographic characteristics of physicians were shown in Table 1.

**Table 1:** Demographic characteristics of physicians

Characteristic		N (%)
Gender	Male	3523 (54.5%)
	Female	2724 (43.6%)
Age	20–30	1349 (21.6%)
	31–40	2255 (36.1%)
	41–50	2112 (33.8%)
	51+	531 (8.5%)
Work experience in equivalent-years	0–4 years	1293 (20.7%)
	5–9 years	1056 (16.9%)
	10–14 years	1006 (16.1%)
	15–19 years	1106 (17.7%)
	20 years and above	1786 (28.6%)

As shown in Table 2, the results suggested that no significant differences were observed in different gender except for competency of interpersonal communication. For the different age groups and years of working experiences, significant differences were found in the importance evaluation of eight dimensions among physicians.

**Table 2:** The comparison of physicians’ competency in the importance evaluation of eight dimensions

	Gender		Age		Work experience	
	t	P	F	P	F	P
1.Clinical skills and patient care	−2.10	0.35	5.59	0.001	4.37	0.002
2.Professionalism	−1.52	0.12	3.03	0.02	2.86	0.022
3.Interpersonal communication	−2.52	0.01	10.10	<0.001	6.90	<0.001
4.Master of medical knowledge	−0.71	0.47	5.90	0.001	3.79	0.004
5.Teamwork	−0.70	0.47	10.46	<0.001	7.84	<0.001
6.Health promotion and disease prevention	−1.71	0.08	14.78	<0.001	11.46	<0.001
7.Information and management	−1.21	0.22	10.75	<0.001	7.57	<0.001
8.Academic research	0.42	0.67	11.39	<0.001	8.66	<0.001

### Exploratory latent class analysis

Based on the importance results of the 103 competency items by physicians in China, the current study selected the average importance scores for each dimension in the questionnaire. The mean and standard deviation of each dimension of physicians’ competency were shown in Table 3. The first three dimensions of importance evaluation were clinical skill and patient care, professionalism, and interpersonal communication.

**Table 3:** Importance evaluation of physicians’ competency in eight dimensions

	Mean	Standard deviation
1.Clinical skills and patient care	4.54	0.42
2.Professionalism	4.50	0.45
3.Interpersonal communication	4.46	0.50
4.Master of medical knowledge	4.42	0.50
5.Teamwork	4.43	0.51
6.Health promotion and disease prevention	4.34	0.57
7.Information and management	4.35	0.53
8.Academic research	4.38	0.58

Table 4 lists the LCA results of the competency indicators for importance evaluation of physicians. By exploratory LCA, one to eight cluster models were fitted. According to the model fit parameter and theory, BIC gradually reduced from the baseline model to the four-class model, and then began to rise with five-class model. Among the eight models, the four-class one is the best model with the smallest BIC value, suggesting satisfactory fitting of the original data. Therefore, the four-class model was chosen as fitting the final model.

**Table 4:** Fitness indicators of different latent class models

	BIC	AIC	df	*P*-value
1-Class	20,258.18	20,204.26	247	<0.001
2-Class	17,428.45	17,313.87	238	<0.001
3-Class	17,275.14	17,099.91	229	<0.001
4-Class	17,238.98	17,003.08	220	<0.001
5-Class	17,241.09	16,944.54	211	0.020
6-Class	17,293.57	16,936.36	202	0.094
7-Class	17,353.99	16,936.12	193	0.180
8-Class	17,418.60	16,940.07	184	0.250

### Latent classification probability and conditional probabilities of the selected model

The four-class LCA chosen as described above was utilized to calculate the corresponding latent classification probability and conditional probabilities. The competency of physicians was evaluated based on their importance evaluation of the eight competency dimensions.

As shown in Table 5, in Class 1, importance evaluation of the physicians suggested eight competency dimensions were all important, which can be determined as “excellent competency” group. In Class 2, importance evaluation of the physicians suggested that seven competency dimensions were important, and the dimension of professionalism was evaluated as unimportant, which was determined as the “lack of professionalism competency” group. In Class 3, importance evaluation of the physicians suggested six competency dimensions were important, but the dimensions of teamwork and academic research were evaluated as unimportant, therefore these physicians were determined to be the “individual competency driven” group. In Class 4, importance evaluation of the physicians suggested the eight competency dimensions were all unimportant, which can be determined to be the “lack of cognitive competency” group.

**Table 5:** Latent classification probability and conditional probabilities of the eight competency dimensions of the physicians

	Conditional probability			
	Class = 1	Class = 2	Class = 3	Class = 4
1				
Unimportant	0.0295	0.3964	0.0763	0.6370
Important	0.9705	0.6036	0.9237	0.3630
2				
Unimportant	0.0325	0.5480	0.2370	0.9795
Important	0.9675	0.4520	0.7630	0.0205
3				
Unimportant	0.0296	0.2672	0.1877	0.8568
Important	0.9704	0.7328	0.8126	0.1432
4				
Unimportant	0.0077	0.1725	0.2484	0.8549
Important	0.9923	0.8275	0.7516	0.1451
5				
Unimportant	0.0053	0.1309	0.3445	0.7875
Important	0.9947	0.8691	0.6555	0.2125
6				
Unimportant	0.0113	0.0592	0.5162	0.8622
Important	0.9887	0.9408	0.4838	0.1378
7				
Unimportant	0.0119	0.1815	0.5510	0.9034
Important	0.9881	0.8185	0.4490	0.0966
8				
Unimportant	0.0065	0.0753	0.3154	0.6168
Important	0.9935	0.9247	0.6846	0.3832
Latent classification probability	0.8801	0.0657	0.0432	0.0110

### Latent class categorization of the physicians’ competency

The final step of the LCA is to categorize all the individuals into appropriate groups of latent classification, and to calculate the probability of dividing the different combinations of eight dimensions into each latent class.

Table 6 lists the categorization results of different combinations of eight dimensions. For example, if a physician evaluated the competency dimensions as “important” in all eight factors, the probability of being categorized into the latent Class 1 is 0.9890, which is higher than the probabilities of other categories, and therefore grouped into latent Class 1. Based on the same theory, 6247 physicians can be divided into four latent classes based on their importance evaluation results of competency dimensions, and the number of each class is 5684, 284, 215 and 64, respectively.

**Table 6:** Individual categorization results of the latent class model

Combination of variables	Frequency	Posterior Probability				Class
{1,2,3, … 7,8}		C1	C2	C3	C4	
{2,2,2,…,2,2}	4854	0.9890	0.0087	0.0023	0	1
{2,2,2,…,2,1}	41	0.7833	0.0858	0.1309	0	1
…	…	…	…	…	…	…
{1,1,1,…,1,2}	10	0	0.0007	0.0021	0.9972	4
{1,1,1,…,1,1}	12	0	0	0.0006	0.9994	4

## Discussion

Currently, the selection, training, and performance evaluation of physicians in China are mainly based on their basic medical knowledge, skills and work performance. Knowledge and skills are easy to be trained, which can be evaluated through examinations. However, the real work performance of many seemingly capable people can turn out to be disappointing. The reason behind such phenomenon may be the ignorance of other competency elements. How to objectively evaluate the physicians, and how to effectively design specific training programs for the physicians, depends on the establishment of a physician competency model with Chinese characteristics [26]. The research on competency models of physicians in China is still at the early stage, which is far from being able to provide a full-range, objective and standard evaluation system for the selection, training and evaluation of physicians, the establishment of the evaluation competency model is a key issue to be solved in our current society. However, before the establishment of the competency evaluation model, we still need to estimate the subgroups of physicians’ competency.

The LCA can be used to measure and analyze a number of abstract indicators that could not be directly observed, which can simplify and integrate complex human behavior experience and social phenomenon data [27]. To our knowledge, no previous studies have identified classes of physicians’ competency. The aim of this study was to identify subgroups of physicians’ competency based upon the importance evaluation of competency questionnaire. The latent clustering numbers were not pre-set before analysis, nor were specific limits set for parameters.

According to the importance evaluation results in eight dimensions, the competency of physicians can be divided into four classes: excellent competency, lack of professionalism competency, individual competency driven, and lack of cognitive competency. The physicians in Class 1, named “excellent competency” group, with competency evaluation of important in eight dimensions. The physicians in this group are equipped with good awareness of competency importance. The physicians in Class 2 mainly lacked awareness of importance of professionalism competency. The professionalism competency emphasized that physicians should regard serving for people’s health as the highest standards of ethics. In the medical career, physicians need to have the ultimate faith, compromising altruism, pursuit of excellence, sincere and trustworthy, strong sense of responsibility. Medical professionalism is the basis for the trust in patient-physician relationship. The view of improving health care through increasing medical professionalism has been gaining momentum among physician organizations [28–30]. Especially in China, physicians find it increasingly difficult to meet their responsibilities to patients and society. In these circumstances, physicians should reaffirm the fundamental and universal principles and values of medical professionalism, which remain ideals to be pursued [31, 32]. Likewise, the medical universities must do more to ensure that medical professionalism among trainees is understood, discussed and practiced.

The physicians in Class 3 lacked awareness of importance of teamwork and academic research competency. The teamwork competency mainly includes good coordination with team members, willingness to help colleagues, and building good relationships with other departments. It is effective to develop a patient’s treatment plan in the form of teamwork, which improves patient safety, increase job satisfaction and maintain the stability of the medical team. Each medical unit has a complete consultation referral system, physicians should maintain an objective and impartial attitude in consultation with other departmental patients, and work out the treatment plan together [33, 34]. Academic research includes reading academic literature, cultivating innovation ability, participating in scientific research, and actively writing research articles. The physicians should take an active part in academic conferences, so as to broaden the professional academic vision and increase the breadth and depth of thinking. Therefore, the health administrative departments should highlight teamwork and academic research training to promote physicians’ competency for this subgroup in the future. In Class 4, the physicians lacked awareness of importance of eight dimensions. Few physicians belong to this subgroup, but in the process of clinical diagnosis and treatment, medical negligence will endanger the patient health [35]. The health administrative departments should emphasize the training and assessment of physicians’ competency to meet the demands of clinical practice especially for this subgroup. Moreover, the health administration could provide detailed training plans for this subgroup, and offering comprehensive assessment in the future training and management.

There are several limitations in our study. Firstly, the cross-sectional design was used in this study, which precluded the analysis of causality. Future longitudinal studies are needed to further explore the relationship between physicians’ competency and their associated factors. Secondly, this study relied on physicians’ importance evaluation data from competency questionnaires in China, although the instruments used were standardized and validated. Future research could benefit from using a more detailed data-collection method such as 360-degree assessment for physicians. Thirdly, the application of the research was not studied, and the hospital should provide individualized training schemes for different physicians’ competency subgroups [36].

## Conclusions

The physicians’ competency can be divided into four distinct subgroups. The LCA approach can provide important information on how interventions could be targeted at or tailored for different physician subgroup. In future training of physicians, the health administrative departments and hospitals could utilize more specific measures according to their different competency subgroups, providing individualized training schemes. At the same time, the competency classification results can provide a categorical basis for evaluation and prediction of competency, providing a good strategic basis for the future training of modern medical talents with rich scientific knowledge, superb clinical ability and comprehensive professional qualities.

## Additional file

Additional file 1: Chinese physician’s competency questionnaire. (DOCX 29 kb)

## Abbreviations

ACGME: Accreditation Council for Graduate Medical Education
AIC: Akaike information criterion
BIC: Bayesian information criterion
CanMEDS: Canadian Medical Education Directives for Specialists
CMB: China Medical Board
GMC: General Medical Council
GMP: Good Medical Practice
LCA: latent class analysis
LCM: latent class model
RCPSC: Royal College of Physicians and Surgeons of Canada
